# Effects of high-intensity interval training on cardiorespiratory fitness, jump, and sprint performance in male under-20 soccer players: a systematic review

**DOI:** 10.3389/fphys.2026.1828517

**Published:** 2026-07-30

**Authors:** Jordan Hernandez-Martínez, Joaquín Perez-Carcamo, Izham Cid-Calfucura, Edgar Vásquez-Carrasco, Sebastian Canales-Canales, Tomás Herrera-Valenzuela, Leonardo Vidal Andreato, Braulio Henrique Magnani Branco, Pablo Valdés-Badilla

**Affiliations:** 1 Department of Physical Activity Sciences, Universidad de Los Lagos, Osorno, Chile; 2 Department of Education, Faculty of Humanities, Universidad de la Serena, La Serena, Chile; 3 Department of Physical Activity, Sports and Health Sciences, Faculty of Medical Sciences, Universidad de Santiago de Chile (USACH), Santiago, Chile; 4 School of Occupational Therapy, Faculty of Psychology, Universidad de Talca, Talca, Chile; 5 Centro de Investigación en Ciencias Cognitivas, Faculty of Psychology, Universidad de Talca, Talca, Chile; 6 VITALIS Longevity Center, Universidad de Talca, Talca, Chile; 7 Graduate Program in Health Promotion, Cesumar University (UniCesumar), Maringá, Brazil; 8 Department of Physical Activity Sciences, Faculty of Education Sciences, Universidad Católica del Maule, Talca, Chile; 9 Sports Coach Career, Faculty of Life Sciences, Universidad Viña del Mar, Viña del Mar, Chile

**Keywords:** athletes, exercise, physical fitness, physiology, sports

## Abstract

**Background and objectives:**

This systematic review and meta-analysis aimed to examine the effects of high-intensity interval training (HIIT) on cardiorespiratory fitness, jump performance and sprint performance in male under-20 soccer players.

**Materials and methods:**

A systematic search was conducted across PubMed, Medline, SportDiscus, CINAHL Complete, Scopus, and Web of Science up to March 2026. The review followed PRISMA guidelines and was prospectively registered in PROSPERO. Eligible studies were randomized controlled trials involving male soccer players younger than 20 years exposed to HIIT interventions. Methodological quality was assessed using the TESTEX scale, risk of bias with RoB 2, and certainty of evidence with GRADE.

**Results:**

Eight randomized controlled trials involving 203 participants were included. Interventions lasted 5 to 12 weeks, with two to three sessions per week. Meta-analysis showed that HIIT significantly improved VO_2_max compared with active comparator/control conditions (Hedges’ g = 0.53; 95% CI: 0.16 to 0.90; p = 0.0284), while no significant effect was observed for Yo-Yo test performance (Hedges’ g = 0.26; 95% CI: −0.58 to 1.09; p = 0.4911). Narrative synthesis showed inconsistent findings for jump and sprint performance, although some studies reported improvements in 30-m sprint performance. The certainty of evidence was low due to serious risk of bias.

**Conclusions:**

HIIT may improve selected physical performance outcomes, particularly VO_2_max and longer sprint performance, in male under-20 soccer players.

**Systematic Review Registration:**

https://www.crd.york.ac.uk/prospero/display_record.php?RecordID=1167616, identifier CRD420251167616.

## Graphical Abstract

**Figure:**
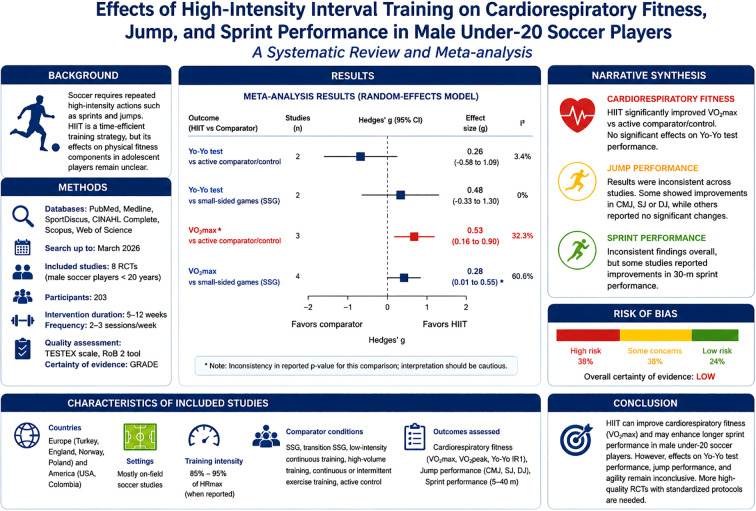
This was created using artificial intelligence.

## 1 Introduction

Soccer is a sport of intermittent, high-intensity actions dominated by sprints in different directions, jump, and ball kicking (Dolci et al., 2020). The ultimate goal of these actions is to score or prevent a goal, depending on the position of play (Hernandez-Martinez et al., 2024). These actions frequently occur during in a soccer game, with many actions performed at intensities above 80% to 90% of maximum heart rate (Dellal et al., 2012; Hostrup and Bangsbo, 2023), interspersed with very short rest periods after each action ranging from 20 to 60 seconds (Selmi et al., 2016). Following high-intensity exercise, the recovery phase is governed by aerobic metabolism, which restores homeostasis by resynthesizing phosphocreatine and clearing accumulated intracellular inorganic phosphate (Pi) and blood lactate (Alghannam, 2012). The duration of high-intensity stimuli and recovery time will depend on the competitive level (amateur, semi-professional, professional, or elite), as well as the age of the competitors (children, adolescents, and adults) (Hostrup and Bangsbo, 2023; Selmi et al., 2016). The effective execution of explosive actions in soccer is, therefore, contingent upon an athlete possessing both a solid aerobic capacity and substantial anaerobic power (Di Giminiani and Visca, 2017).

High-intensity interval training (HIIT) presents a time-efficient alternative to conventional training, effectively inducing physiological adaptations relevant to soccer (Kunz et al., 2019). HIIT is commonly defined as repeated short to long bouts of high-intensity exercise interspersed with recovery periods, with interval formats ranging from short efforts <45 s to longer bouts of approximately 2–4 min (Buchheit and Laursen, 2013b). HIIT should not be considered a single uniform method, as long intervals, short intervals, sprint interval training, repeated sprint training, and game-based formats such as small-sided games may induce different cardiorespiratory, metabolic, and neuromuscular responses (Buchheit and Laursen, 2013a). These adaptations may occur through both central and peripheral mechanisms. At the central level, HIIT may improve maximal oxygen uptake (VO_2_max) by increasing stroke volume, maximal cardiac output, and oxygen delivery, whereas peripheral adaptations may include greater skeletal muscle oxidative capacity, mitochondrial content, oxygen extraction, phosphocreatine resynthesis, buffering capacity, and fatigue resistance during repeated high-intensity efforts (Buchheit and Laursen, 2013a; MacInnis and Gibala, 2017). These mechanisms are particularly relevant in soccer, where players are required to repeatedly perform high-intensity running, sprinting, accelerations, decelerations, and short recovery periods during match play (Buchheit and Laursen, 2013a; MacInnis and Gibala, 2017). Which has demonstrated improvements in aerobic capacity (Clemente et al., 2021a), and power-based actions (Boraczyński et al., 2023; Yang et al., 2024) in soccer players. These results are reported in a meta-analysis conducted by Clemente et al. (2021a) on amateur, semi-professional, and professional soccer players, both young and adult, reporting significant improvements in maximum oxygen consumption (VO_2_max) in favor of HIIT compared to active control groups. In addition, no significant differences in repeated sprint ability, jump performance, and linear sprinting time were found. However, in a systematic review with meta-analysis conducted by Clemente et al. (2021b) on young university players, HIIT was compared with small-sided games, with no significant differences reported in repeated sprint ability among soccer players at this level. In contrast, a meta-analysis conducted by Kunz et al. (2019) on young soccer players reported significant improvements in VO_2_max, repeated sprint capacity, and lactate threshold with both HIIT and small-sided games in players at this level. Previous systematic reviews have examined the effects of HIIT on physical fitness outcomes in male soccer players across different competitive levels and age groups (Manuel Clemente al., 2021).

Importantly, youth soccer players should not be considered as smaller versions of adult athletes. During adolescence and the transition to early adulthood, substantial physiological, hormonal, neuromuscular, and morphological changes occur that may influence both physical performance and responsiveness to training stimuli (Lloyd and Oliver, 2012; Pichardo et al., 2018). Factors such as biological maturation, growth-related changes in body composition, endocrine development, and improvements in neuromuscular coordination can substantially modify the magnitude and nature of training adaptations (Lloyd and Oliver, 2012; Pichardo et al., 2018). Consequently, evidence derived from senior soccer players cannot be directly extrapolated to younger populations, as similar training stimuli may elicit different physiological and performance responses depending on maturation status and developmental stage. Therefore, a population-specific synthesis is needed to better understand the effects of HIIT in male under-20 soccer players.

Thus, although there is evidence on the effects of HIIT on soccer players of different levels and ages, these results are unclear regarding the effect of HIIT (Clemente et al., 2021b; Kunz et al., 2019; Clemente et al., 2021a). It is important to consider that physical performance and the response to training can differ according to the age of both young and adult players (Clemente et al., 2021b). Considering that it is necessary to update the evidence in sports science (Beato et al., 2025), as well as the importance of conducting training focused on improving specific actions in formative ages in soccer (Práxedes et al., 2017). In this sense, this systematic review with meta-analysis aimed to analyze the effect of HIIT on cardiorespiratory fitness, jump, and sprint performance in male adolescents’ soccer players.

## 2 Methods

### 2.1 Protocol and registration

The PRISMA guidelines were followed in this systematic review (Page et al., 2021). PROSPERO (the International Prospective Register of Systematic Reviews; ID code: CRD420251167616) has the protocol registered.

### 2.2 Eligibility criteria

Eligibility criteria followed the PICOS framework (Liberati et al., 2009). Inclusion and Exclusion Criteria To be eligible for inclusion, studies were required to: (a) involve participants younger than 20 years; (b) utilize a high-intensity interval training (HIIT) protocol; (c) incorporate an active control group; (d) assess at least one pre- and post-intervention outcome related to cardiorespiratory fitness, jump performance or sprint performance; and (e) be a peer-reviewed, randomized controlled trial.

Studies were subsequently excluded for any of the following reasons: (1) lack of reportable pre- and post-intervention data for qualitative synthesis; (2) use of a multicomponent intervention that prevented the isolation of HIIT effects; (3) significant methodological limitations compromising interpretation of results (e.g., flawed randomization); (4) data duplication from a previously included publication; or (5) critically low methodological quality score. From the outset, non-original articles, such as reviews, editorials, letters, conference abstracts, books or book chapters, and non-randomized designs were not considered for inclusion (see **Table 1**).

**Table 1:** Selection criteria used in the systematic review.

Category	Inclusion criteria	Exclusion criteria
Population	Male soccer players 12 to <20 years of age, with no restrictions on competitive level or age.	Female soccer players, male adult soccer players or players < 20 years of age with health problems (e.g., injuries, recent surgery) and soccer players.
Intervention	isolated (i.e., not combined with other methods) soccer-specific (i.e., running-based). HIIT interventions with a minimum duration of at least 2 weeks, with intermittent episodes performed at ≥85–90% of maximum heart rate, ≥90% of the speed associated with VO_2_max or near-maximal/maximal sprint efforts, also with no restrictions for duration (e.g., short-intervals ≤60 seconds per bout, long-interval HIIT >60 seconds, small-sided games, speed endurance training, repeated sprint training and sprint interval training).	HIIT applied to other sports, used HIIT combined with other training methods, used combined HIIT types (e.g., running-based long-interval HIIT combined with small-sided games), used other than running-based HIIT (e.g., cycling, boxing, rowing).
Comparator	Active control group.	Absence of active control group
Outcome	At least one physical fitness measure of muscle power (i.e., jump performance), cardiorespiratory fitness (i.e., VO_2_max, yoyo test), sprint speed performance before and after the training intervention.	Lack of baseline and/or follow-up data
Study design	Randomized controlled trials	Non-randomized controlled trials.

### 2.3 Information search process and databases

The search process was conducted between September 2024 and March 2026 across six general databases: PubMed, Medline, SportDiscus, CINAHL Complete, Scopus, and Web of Science (core collection). The search strategy was adapted to each database. The overall search string was: (“football”) AND (“high-intensity interval training” OR “HIIT” OR “high-intensity intermittent training” OR “interval training” OR “small-sided games” OR “sprint interval training” OR “repeated sprint training” OR “speed endurance training”) AND (“physical fitness” OR “physical performance” OR “conditional performance” OR “agility” OR “speed” OR “sprint” OR “reaction time” OR “coordination” OR “endurance” OR “strength endurance” OR “vertical jump” OR “countermovement jump” OR “high jump” OR “reactive strength”) AND (“teenager” OR “teenagers” OR “adolescence” OR “teenagers” OR “teenager” OR “teenagers” OR “young” OR “young athlete” OR “young athletes” OR “youth football” OR “academia” or “junior”).The search was supplemented by consultation with two independent experts, selected based on their academic background (PhD in sport science) and publication history (peer-reviewed articles on physical performance in journals indexed in the JCR^®^). To ensure impartial input, these experts were blinded to the primary search strategy. As a final quality control measure, the list of included publications was checked against major databases on March 1, 2026, to detect any subsequent retractions or errors.

### 2.4 Study selection and data collection process

The EndNote reference manager (version X9, Clarivate Analytics, Philadelphia, PA, United States of America) exported the studies. JHM and PVB conducted separate searches, eliminated duplicates, examined titles and abstracts, and examined complete texts. At this point, no disparities were discovered. The procedure was repeated for recommendations made by outside specialists and searches inside reference lists. The texts of possibly suitable papers were then examined, and the rationale behind excluding those not fitting the selection criteria was disclosed.

### 2.5 Methodological quality assessment

TESTEX, a tool for exercise-based intervention studies, was used to assess the methodological quality of the chosen studies (Smart et al., 2015). According to Smart et al. (2015), there is a 15-point rating system (five points for study quality and 10 points for reporting). Two authors (JHM, PVB) carried out this process separately, whereas a third author (ICC) served as a referee for cases that were borderline and needed further validation from another author (THV).

### 2.6 Data synthesis

The following data were obtained and analyzed from the selected studies: (i) author and year of publication; (ii) study design; (iii) number participants and country; (iv) competitive level; (v) number of participants in the intervention and control group (CG); (vi), mean age of the sample; (vii) activities performed in the HIIT and CG; (viii) settings; (ix) training intensity; (x) training volume (total duration, weekly frequency and time per session); (xi) training protocol; (xii) cardiorespiratory fitness; (xiii) jump performance; (xiv) sprint performance; and (xv) results outcome. A qualitative synthesis of the findings was performed, grouping studies according to the main physical fitness outcomes in cardiorespiratory fitness using both continuous protocols (VO_2_max) and intermittent protocols (Yo-Yo IR1), as well as sprint performance (0–10 m, 0–20 m, 0–30 m, and 0–40 m sprints) and jump performance (CMJ, SJ, and DJ). For cardiorespiratory fitness outcomes, we extracted whether VO_2_max or $\dot{V}$O_2_peak was directly measured using laboratory-based protocols or indirectly estimated from field-based tests. Yo-Yo IR1 and Course Navette outcomes were treated as field-based estimates or indicators of intermittent aerobic fitness rather than direct VO_2_max measurements. Due to methodological heterogeneity across studies, outcomes that could not be quantitatively pooled were summarized narratively. When at least two studies reported comparable outcomes, data were synthesized using meta-analysis.

### 2.7 Risk of bias in individual studies

Two independent researchers (JHM and ICC) evaluated the risk of bias version 2 (RoB 2) of the included studies, and a third researcher (PVB) analyzed the results. The Cochrane Handbook for Systematic Reviews of Interventions’ recommendations for RCTs was the foundation for this evaluation (Sterne et al., 2019). On the basis of the randomization procedure, departures from the planned interventions, missing outcome data, outcome assessment, and choice of the reported result, the risk of bias was categorized as “high”, “low”, or “some concerns”.

### 2.8 Measures for meta-analysis

The study methodology includes a meta-analysis, with full information available on PROSPERO (registration code: CRD420251167616). The standardized mean difference (SMD), a statistic that assesses the absolute difference between mean values in two groups in a randomized controlled trial, was calculated for each analysis using Rstudio Software (version 2026.04.0), with a p value of <0.05 considered statistically significant (Higgins and Green, 2009). In each trial, the random-effects model (Der Simonian–Laird approach) was employed to calculate and pool the SMD and mean difference (MD) of HIIT function from pre-intervention to post-intervention, comparing experimental and control groups or other groups (Davey et al., 2011). The fundamental premise of the random-effects model is that true effects (e.g., interventions, duration) vary across studies, with samples drawn from populations with different effect sizes. If at least two studies yielded consistent results, the data were pooled. Heterogeneity between trial results was tested using the Cochran Q test (Morris, 2008) and the I^2^ statistic. I^2^ values of <25%, 25–50%, and >50% indicate small, medium, and large inconsistencies, respectively (Higgins et al., 2003). Egger regression tests were also performed to detect small study effects and potential publication bias (Higgins and Thompson, 2002).

### 2.9 Certainty of evidence

Studies were categorized as having high, moderate, low, or very low confidence on the basis of their assessment of the GRADE scale (Guyatt et al., 2011). Because studies with RCT designs were included, all analyses began with a high degree of certainty and were downgraded if there were concerns about bias, consistency, accuracy, precision, directness of results, or risk of publication bias (Guyatt et al., 2011). Two authors evaluated the studies separately (JHM, PVB), and any disagreements were settled by agreement with a third author (THV).

## 3 Results

### 3.1 Study selection

**Figure 1:**
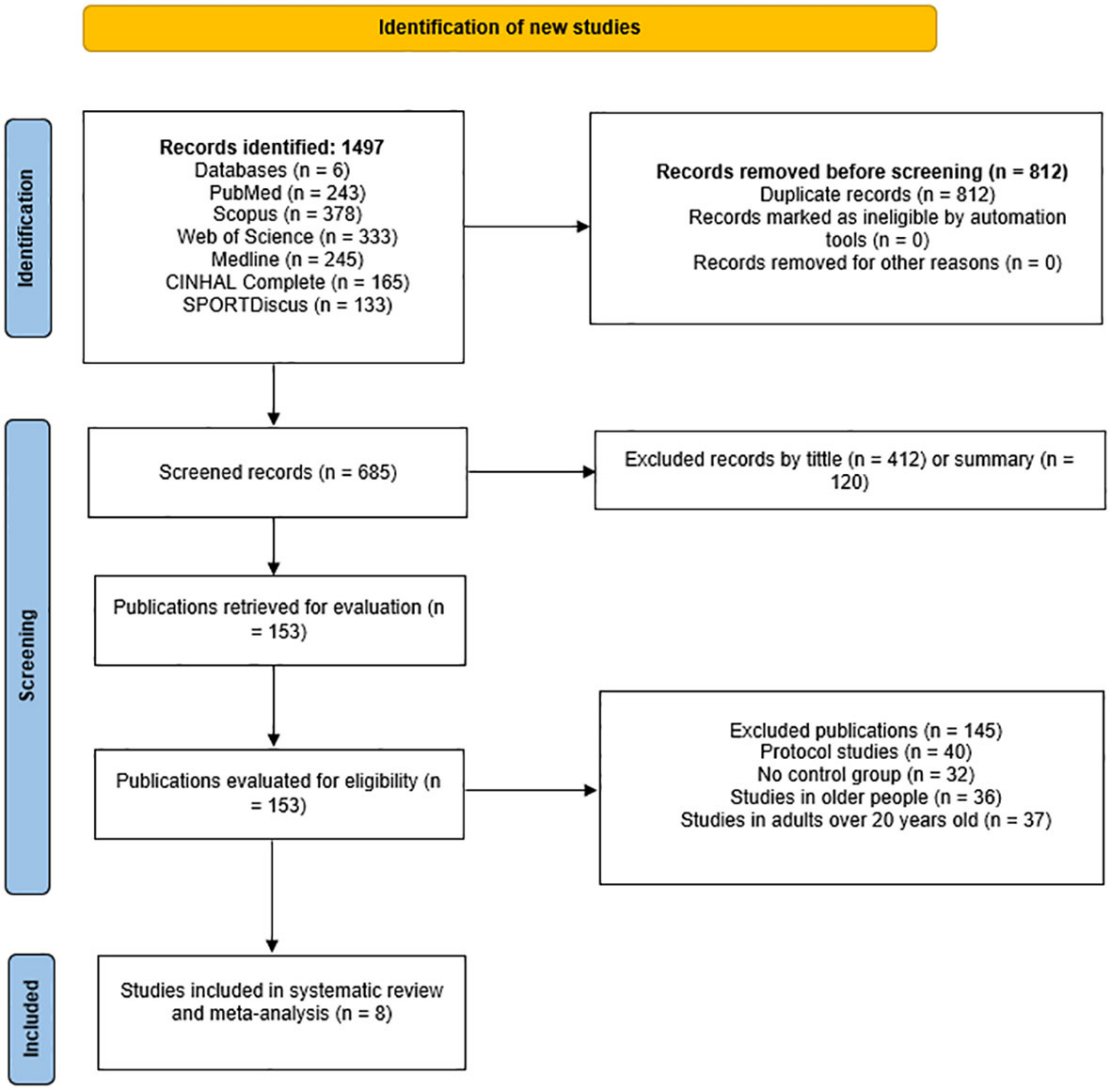
Flowchart of the review process. Legends: Based on the PRISMA guidelines (Page et al., 2021).

A total of 1,497 records were found in six databases. Subsequently, duplicates were removed and studies were filtered by selecting the title, abstract, and keywords, resulting in 685 references. In the subsequent analysis phase, 532 articles were excluded because the texts did not meet the search criteria, leaving 153. Subsequently, 40 protocol studies, 32 without a control group, 36 studies in older people, and 37 studies in adults over 20 years were excluded. This left eight articles that met the inclusion criteria (Arslan et al., 2020; Böge and Patlar, 2022; Helgerud et al., 2001; Howard and Stavrianeas, 2017; Jastrzębski et al., 2014; Runacres et al., 2019; Salazar et al., 2023; Sperlich et al., 2011). These results are presented in the flowchart (**Figure 1**). The included studies involved 203 soccer players under 20 years old as participants, with a mean age of 14.95 years. The characteristics of the participants and the HIIT interventions used in the included studies are shown in **Table 2**.

**Table 2:** Study quality assessment according to the TESTEX scale.

Eligibility criteria specified	Randomly allocated participants	Allocation concealed	Groups similar at baseline	Assessors blinded	Outcome measures assessed >85% of participants *	Intention to treat analysis	Reporting of between group statistical comparisons	Point measures and measures of variability reported **	Activity monitoring in control group	Relative exercise intensity reviewed	Exercise volume and energy expended	Overall TESTEX#
Arslan et al. (2020)	Yes	No	Yes	No	Yes (1)	No	Yes	Yes (2)	Yes	Yes	Yes	9/15
Böge and Patlar (2022)	Yes	No	Yes	No	Yes (1)	No	Yes	Yes (2)	Yes	Yes	Yes	9/15
Helgerud et al. (2001)	Yes	No	Yes	No	Yes (1)	No	Yes	Yes (2)	Yes	Yes	Yes	9/15
Howard and Stasinos Stavrianeas (2017)	Yes	No	Yes	No	Yes (1)	No	Yes	Yes (2)	Yes	Yes	Yes	9/15
Jastrzębski et al. (2014)	Yes	No	Yes	No	Yes (1)	No	Yes	Yes (2)	Yes	Yes	Yes	9/15
Runacres et al. (2019)	Yes	No	Yes	No	Yes (1)	No	Yes	Yes (2)	Yes	Yes	Yes	9/15
Salazar et al. (2023)	Yes	Yes	Yes	Yes	Yes (1)	No	Yes	Yes (2)	Yes	Yes	Yes	11/15
Sperlich et al. (2011)	Yes	No	Yes	Yes	Yes (1)	No	Yes	Yes (2)	Yes	Yes	Yes	9/15

*Three points are possible: one point if adherence >85%, one point if adverse events were reported, and one point if exercise attendance was reported. **Two points possible: one point if the primary outcome is reported and one point if all other outcomes are reported. # Total out of 15 points. TESTEX: Tool for assessing study quality and reporting in exercise.

### 3.2 Methodological quality

The 8 selected studies were analyzed via the TESTEX scale (**Table 2**). All studies scored 60% or higher on the TESTEX scale (Arslan et al., 2020; Böge and Patlar, 2022; Helgerud et al., 2001; Howard and Stavrianeas, 2017; Jastrzębski et al., 2014; Runacres et al., 2019; Salazar et al., 2023; Sperlich et al., 2011), namely 9/15 (Arslan et al., 2020; Böge and Patlar, 2022; Helgerud et al., 2001; Howard and Stavrianeas, 2017; Jastrzębski et al., 2014; Runacres et al., 2019; Sperlich et al., 2011), and one study 11/15 (Salazar et al., 2023).

### 3.3 Data synthesis

The eight randomized controlled trials included a total of 203 male soccer players, with mean ages ranging from approximately 14.1 to 18.1 years. The studies were conducted across different continents, including Europe (Turkey, England, Germany, Norway, and Poland) and the Americas (United States of America and Colombia). The intervention duration ranged from 5 to 12 weeks, with a weekly frequency of two to three sessions. HIIT protocols varied substantially across studies, including short-interval running formats, repeated sprint protocols, 4 × 4 min intervals, and soccer-specific drills, generally performed at high intensities ranging from 85% to 95% of maximal heart rate when reported. Comparator conditions included small-sided games, transition small-sided games, low-intensity continuous training, high-volume training, continuous or intermittent exercise training, and active control groups. The assessed outcomes included cardiorespiratory fitness, jump performance, and sprint performance. These characteristics are presented in **Table 3**.

**Table 3:** Characteristics of participants examined in the included studies.

Authors	Study design	Sample size/group	Country	Competitive level	Age years	Setting	Training Intensity	Intervention duration (weeks)	Frequency (sessions/week)	Session duration (min)	Training Protocol	Cardiorespiratory fitness assessment protocol	Cardiorespiratory fitness outcome	Jump performance	Sprint performance	Results
Arslan et al. (2020)	RCT	HIIT: 10 SSG: 10	Turkey	NR	HIIT: 14.1 ± 0.6 SSG: 14.4 ± 0.5	Football field with synthetic turf	90%–95% HRmax	5	2	HIIT: 12-20 SSG: 10-18	HIIT: 2 sets of (6 min of 15”-15”) with progressive increases in working time throughout the weeks. SSG: 2 sets of (2 x 2.30 min) with progressive increases in working time throughout the weeks.	YYIRTL-1; VO_2_max estimated using: VO_2_max = 36.4 + 0.0084 × distance.	(Yo-Yo IR1 performance) VO_2_max (ml. min-1.kg-1)	CMJ SJ, DJ	Sprint (10 and 30 m)	HIIT ↑VO_2_max (ml. min-1.kg-1) ↑ (Yo-Yo IR1 performance) ↑CMJ ↑SJ ↑DJ ↑10 m ↑20 m ↑30 m SSG ↑VO2max (ml. min-1.kg-1) ↑ (Yo-Yo IR1 performance) ↑CMJ ↑SJ ↑DJ ↑10 m ↑20 m ↑30 m
Böge and Patlar (2022)	RCT	HIIT: 8 SSG: 8 TSSG: 8 LICT: 8 CG: 8	Turkey	NR	16.7 ± 1.1	Grass field	HIIT: 90-95% HRmax and 70% HRmax SSG: NR TSSG: NR LICT: 70% HRmax CG: NR	8	3	HIIT: ~16 SSG: 12 TSSG: 12 minutes LICT: 45 minutes	HIIT: 3 sets of (8 repetitions of 15” work/15” rest). SSG: 3 sets of 4 minutes of 4v4 games. TSSG: 3 sets of 4 minutes of 4v4 transition games. LICT: 45 minutes of continuous running CG: routine football training	Yo-Yo IR1; maxVO_2_ estimated using: distance × 0.0084 + 36.4.	(Yo-Yo IR1 performance)	NR	NR	HIIT ↑(Yo-Yo IR1 performance) TSS ↑(Yo-Yo IR1 perfoamance) LICT ↑(Yo-Yo IR1 performance) CG ↑(Yo-Yo IR1 performance) SSG ↔(Yo-Yo IR1 performance)
Helgerud et al. (2001)	RCT	HIIT: 9 CG: 10	Norway	Elite	18.1 ± 0.8 years	Treadmill (for tests) and soccer field (for match analysis)	90-95% HRmax: 50-60% HRmax	8	2	28	HIIT: HIIT 4 x 4 minutes CG: Additional technical training (heading drills, free kicks, ball reception and changes of direction)	Direct treadmill VO_2_max test with gas-exchange analysis until exhaustion.	VO_2_max (ml. min-1.kg-1)	NR	Sprint (10 and 40 m)	HIIT ↑VO_2_max (ml. min-1.kg-1) ↔sprint 10 m ↔sprint 40 m CG ↔VO_2_max (ml. min-1.kg-1) ↔sprint 10 m ↔sprint 40 m
Jastrzębski et al. (2014)	RCT	HIIT: 11 SSG: 11	Poland	NR	HIIT: 15.8 ± 0.6 SSG: 15.8 ± 0.6	Soccer field (training and tests) and Laboratory (Wingate and VO__2_max_ tests)	85–95 HRmax	8	2	HIIT: 21 minutes (7 intervals of 3 minutes) SSG: 21 minutes (7 intervals of 3 minutes)	HIIT: 7 x 3 minutes, 5 seconds of high-intensity running + 15 seconds of jogging SSG: 3 vs 3 games without goalkeepers	Direct graded cycle-ergometer VO_2_max test until exhaustion.	VO_2_max (ml. min-1.kg-1)	NR	Sprint (5 and 30 m)	HIIT ↔VO_2_max (ml. min-1.kg-1) ↔sprint 5 m ↔sprint 30 m SSG ↑VO_2_max (ml. min-1.kg-1) ↔sprint 5 m ↔sprint 30 m
Howard and Stavrianeas (2017)	RCT	HIIT: 16 CG: 16	United States	High School Junior Varsity soccer players	HIIT: 15.1 ± 0.8 CG: 14.8 ± 1.2	Field-based (training and testing)	NR	10	3	HIIT: 4–6 x 30-second sprints (Total high-intensity time: 2–3 minutes per session) CG: Endurance running for the same total duration as the HIIT session.	HIIT: 30-second sprints with progressive increases throughout the weeks with 4.5 minutes of active recovery.CG: Continuous endurance race	Yo-Yo IR1; field-based aerobic conditioning test, no direct VO_2_max.	(Yo-Yo IR1 performance)	CMJ	NR	HIIT ↑(Yo-Yo IR1) ↑CMJ CG ↑(Yo-Yo IR1 performance) ↔CMJ
Runacres et al. (2019)	RCT	HIIT: 14 CIET: 12 CG: 11	England	NR	HIIT: 14.3 ± 3.1 CIET: 13.1 ± 2.5 CG: 13.7 ± 3.2	Grass field	NR	12	3	40	HIIT: Specific football exercises, small-sided games and a full match in the last 15 minutes. CIET: ~30 minutes of continuous running at alternating speeds. CG: healthy children who did not participate in any extracurricular physical activity outside of mandatory physical education lessons within the school.	Direct treadmill ramp test; breath-by-breath gas exchange; peak $\dot{V}$O_2_ reported.	$\dot{V}$O_2_peak absolute	NR	Sprint (30 m)	HIIT ↑ $\dot{V}$O_2_peak absolute ↔sprint 30 m CIET ↔ $\dot{V}$O_2_peak absolute ↔sprint 30 m CG ↔ $\dot{V}$O_2_peak absolute ↔sprint 30 m
Salazar et al. (2023)	RCT	HIIT: 12 SSG: 12	Colombia	NR	NR	Grass field	85% HRmax	9	2	12-30	HIIT: 4 x 3 min work with intervals of 10s and 20s/3 min rest with progressive increase through the weeks. SSG: 2 vs. 2; 4 × 3 min work/3 min rest with progressive increase through the weeks.	Pretest: direct ergospirometry; post-test: indirect Course Navette estimation.	VO__2_max_	NR	NR	HIIT ↔VO_2_max (ml. min-1.kg-1) SSG ↔ VO2max (ml. min-1.kg-1)
Sperlich et al. (2011)	RCT	HIIT: 9 High Volume Training Group: 8	Germany	Youth elite	13.5 ± 0.4	Outdoor court and track training; laboratory evaluations under controlled conditions (20–22 °C, 45–55% RH)	90–95% HRmax	5	3	HIIT: approximately 30 minutes High Volume Training Group: 45–60 minutes per session.	HIIT: 4x4 min intervals, sprints, distances (200m, 400m, 800m). Intensity: 90-95% of maximum heart rate. High Volume Training Group: Continuous running and Fartlek	Direct treadmill VO_2_max test; breath-by-breath gas exchange; highest 30-s value used.	VO_2_max (ml. min-1.kg-1)	DJ SJ CMJ	20-m Sprint (s) 30-m Sprint (s) 40-m sprint (s)	HIIT ↑ VO_2_max (ml. min-1.kg-1) ↑sprint 20-m ↑sprint 30-m ↑sprint 40-m HVTG ↔VO_2_max (ml. min-1.kg-1) ↑sprint 20-m ↑sprint 30-m ↑sprint 40-m

RCT, randomized controlled trial; HIIT, high-intensity interval training; CG, control group; SSG, small-sided games; TSSG, transition small-sided games; LICT, low-intensity continuous training; CIET, continuous and intermittent exercise training; HVTG, high-volume training group; VO_2_max, maximal oxygen uptake; $\dot{V}$O_2_peak, peak oxygen uptake; HRmax, maximal heart rate; Yo-Yo IR1, Yo-Yo Intermittent Recovery Test Level 1; CMJ, countermovement jump; SJ, squat jump; DJ, drop jump; NR, not reported; y/o, years old; min, minutes; m, meters; s, seconds; ↑, significant improvement; ↔, no significant change.

### 3.4 Risk of bias

**Figure 2:**
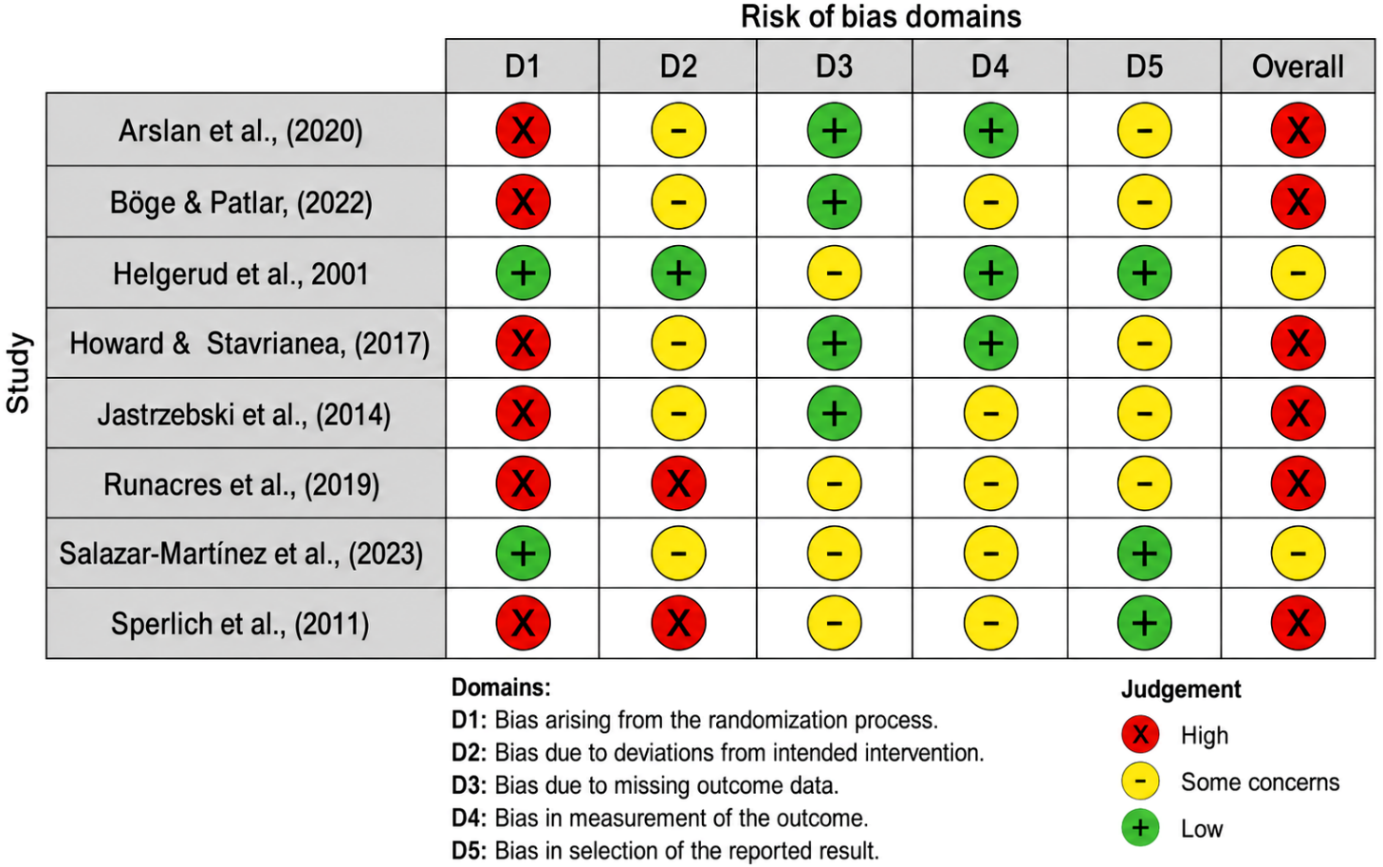
Risk of bias within studies. Legends: D1: randomization process; D2: deviations from the intended interventions; D3: missing outcome data; D4: measurement of the outcome; D5: selection of the reported result.

**Figure 3:**
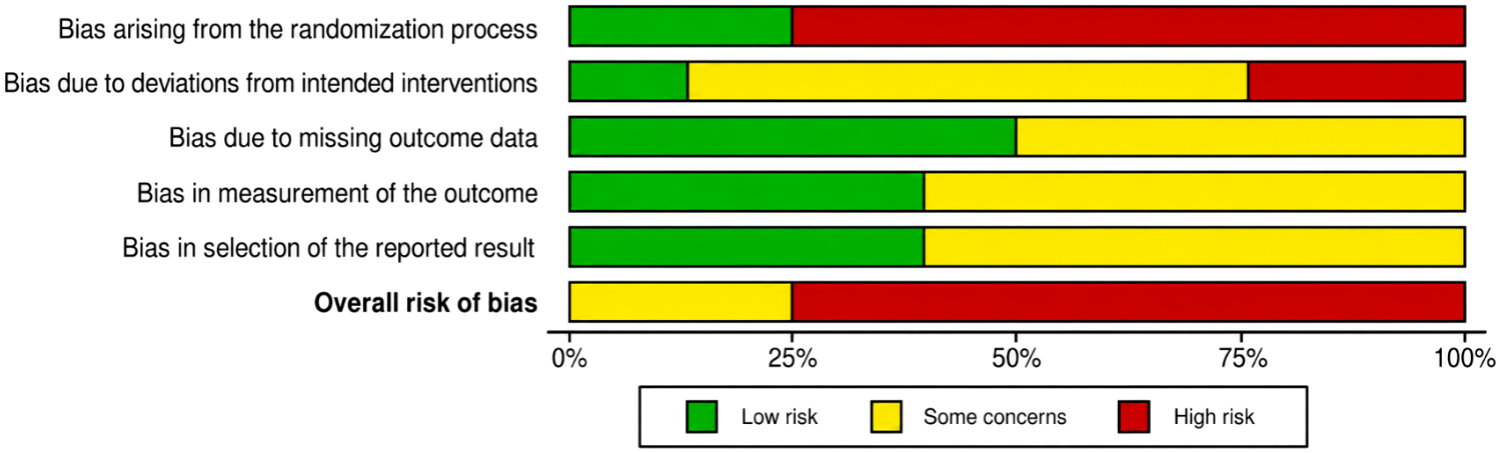
Risk of bias summary: Review the authors; judgments about each risk of bias item in each included study.

Six studies were classified as having a high risk of bias (Arslan et al., 2020; Böge and Patlar, 2022; Howard and Stavrianeas, 2017; Jastrzębski et al., 2014; Runacres et al., 2019; Sperlich et al., 2011), while two study was rated as having some concerns (Helgerud et al., 2001; Salazar et al., 2023). These findings suggest that, although the results provide useful information, the internal validity of these studies is limited due to the high risk of bias (**Figures 2**, **3**).

### 3.5 Outcomes

#### 3.5.1 Jump performance

This was examined in only a limited number of studies just three so these results should be interpreted with caution. Arslan et al. (2020) reported significant improvements in CMJ, SJ, and DJ in both HIIT and SSG groups, while N. Howard and Stavrianeas (2017) observed a significant improvement in CMJ only in the HIIT group. However, B. Sperlich et al. (2011) found no significant changes in DJ, SJ, or CMJ following either HIIT or high-volume training. Therefore, although some studies showed favorable changes, the overall evidence does not consistently support a clear effect of HIIT on jump performance in male adolescent soccer players.

#### 3.5.2 Sprint performance

Five studies were reported for this variable; however, the study designs were highly heterogeneous, with different control groups as well as other experimental groups that differed from HIIT. Arslan et al. (2020) reported significant improvements in performance in 10-m, 20-m, and 30-m sprints in both the HIIT and SSG groups, while Sperlich et al. (2011) found significant improvements in performance in 20 m, 30 m, and 40 m sprints in both the HIIT group and the high-volume training group. However, 3 studies (Helgerud et al., 2001; Jastrzebski et al., 2014; Runacres et al., 2019), did not report significant improvements in sprint performance following HIIT or the comparison training. Therefore, although some studies showed favorable changes, the evidence does not consistently support a clear effect of HIIT on sprint performance in adolescent male soccer players.

#### 3.5.3 Cardiorespiratory fitness

**Figure 4:**
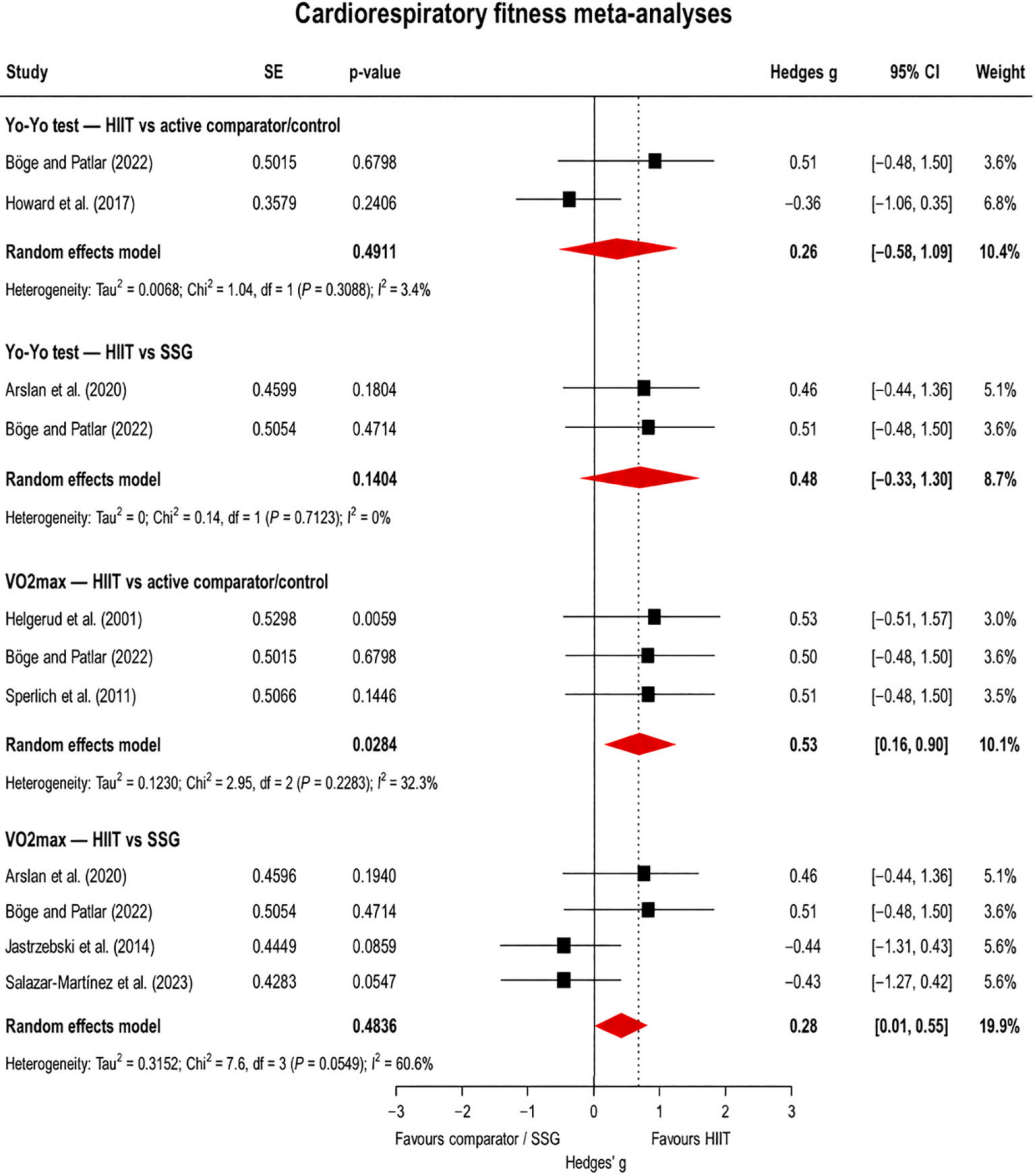
Cardiorespiratory fitness meta-analyses in male under-20 soccer players. Forest plot showing subgroup meta-analyses for Yo-Yo test performance and VO_2_max according to comparator condition: HIIT versus active comparator/control and HIIT versus SSG. Effect estimates are presented as Hedges’ g with 95% confidence intervals. Black squares represent individual study effects, and red diamonds represent pooled random-effects estimates. Positive values favor HIIT, whereas negative values favor the comparator condition or SSG. SE, standard error; CI, confidence interval; HIIT, high-intensity interval training; SSG, small-sided games; VO_2_max, maximal oxygen uptake.

For VO_2_max, HIIT showed a significant positive effect compared with active comparator/control conditions (Hedges’ g = 0.53; 95% CI: 0.16 to 0.90; p = 0.0284; I² = 32.3%). In the comparison between HIIT and SSG, the pooled estimate favored HIIT (Hedges’ g = 0.28; 95% CI: 0.01 to 0.55; I² = 60.6%). However, in the Yo-Yo test, HIIT showed no significant effects compared to the control or active comparison conditions (Hedges’ g = 0.26; 95% CI: −0.58 to 1.09; p = 0.4911; I² = 3.4%) and compared to SSG (Hedges’ g = 0.48; 95% CI: −0.33 to 1.30; p = 0.1404; I² = 0%). These results are shown in **Figure 4**.

### 3.6 Certainty of evidence

Although all evaluated outcomes (cardiorespiratory fitness, jump performance and sprint performance) were derived from randomized controlled trial, the overall certainty of evidence was rated as moderate due to the very serious risk of bias identified across studies. The majority of trials had methodological flaws which led to a decrease of trust in the derived estimated.

There were no significant issues identifies with indirectness; however, some differences were noted in HIIT protocols, outcome measures, and participant characteristics, which may influence the agreement of the results. Besides that, the relatively low number of studies for certain outcomes restricts the accuracy of the present evidence base. Generally, the main point shows that HIIT workouts may positively affect the elements of physical performance in young soccer players. Still, the strength of the evidence should be cautiously understood because of the methodological problems and the variety of the studies involved. All the outcomes were considered as important for the evaluation of physical performance (**Table 4**).

**Table 4:** Summary of findings and certainty of evidence according to GRADE.

Certainty assessment							Number of participants		Effect		Certainty	Importance
Number of studies	Study design	Risk of bias	Inconsistency	Indirectness	Imprecision	Other considerations	HIIT	Control	Relative (95% CI)	Absolute (95% CI)	Certainty	Importance
Cardiorespiratory fitness												
6	randomized trials	very serious^a^	not serious	not serious	not serious	none	66/129 (51.2%)	63/129 (48.8%)	not estimable	not estimable	⊕⊕○○ Low	IMPORTANT
Jump performance												
5	randomized trials	very serious^a^	not serious	not serious	not serious	none	58/109 (53.2%)	51/109 (46.8%)	not estimable	not estimable	⊕⊕○○ Low	IMPORTANT
Sprint performance												
4	randomized trials	very serious^a^	not serious	not serious	not serious	none	56/110 (50.9%)	54/110 (49.1%)	not estimable	not estimable	⊕⊕○○ Low	IMPORTANT
Agility performance												
2	randomized trials	very serious^a^	not serious	not serious	not serious	none	29/56 (51.8%)	27/56 (48.2%)	not estimable	not estimable	⊕⊕○○ Low	IMPORTANT

GRADE, Grading of Recommendations Assessment, Development and Evaluation; RCTs, randomized controlled trials; HIIT, high-intensity interval training; CI, confidence interval; n, number of participants. Certainty symbols: ⊕⊕○○ = low certainty. ^a^ Downgraded two levels because of very serious risk of bias across the included studies. ^b^ Relative and absolute effects were not estimable because the outcomes were synthesized narratively rather than quantitatively pooled.

## 4 Discussion

This systematic review intended to determine the effect of high-intensity interval training (HIIT) on the key performance indicators (cardiorespiratory fitness, jump and sprint performance) of adolescent male football (soccer) players. Generally, the evidence at hand indicates that HIIT might lead to selected physical performance outcomes being elevated; nonetheless, studies’ findings were not consistent. Some studies reported that sprint performance over longer distances improved, while it was more variable and not necessarily in favor of HIIT for the cases of cardiorespiratory fitness, jump performance and short-distance sprint.

### 4.1 Cardiorespiratory fitness

The present systematic review and meta-analysis demonstrated that HIIT was associated with significant improvements in VO_2_max compared with active comparator and control conditions in male under-20 soccer players. In contrast, no significant effects were observed for Yo-Yo test performance. These findings indicate that the available evidence supports a positive effect of HIIT on maximal aerobic power, whereas the evidence for intermittent field-based endurance performance remains less consistent. The improvement in VO_2_max observed in the present review is consistent with previous meta-analyses conducted in broader soccer populations, including youth, amateur, and professional players (Kunz et al., 2019; Clemente et al., 2021a). In this sense, HIIT has been associated with a range of central and peripheral physiological adaptations related to aerobic fitness, including changes in cardiovascular function, oxygen transport, and skeletal muscle oxidative characteristics (Buchheit and Laursen, 2013a; MacInnis and Gibala, 2017). Although these mechanisms were not directly assessed in the studies included in the present review, they have been proposed as potential explanations for the improvements in VO_2_max commonly reported following HIIT interventions (Buchheit and Laursen, 2013a).

Interestingly, these positive adaptations in VO_2_max were not accompanied by significant improvements in Yo-Yo test performance. Although both outcomes are commonly used to evaluate cardiorespiratory fitness, they assess different physiological constructs. VO_2_max primarily reflects maximal aerobic power under continuous exercise conditions, whereas performance in the Yo-Yo test may also be influenced by factors such as repeated acceleration and deceleration ability, change-of-direction capacity, anaerobic contribution, pacing strategies, and technical efficiency (Boraczyński et al., 2023). Consequently, improvements in aerobic capacity do not necessarily translate into proportional gains in intermittent field-test performance. Differences in cardiorespiratory fitness assessment protocols may partly explain the heterogeneity across studies. Some studies directly measured VO_2_max or $\dot{V}$O_2_peak using laboratory gas-exchange protocols, whereas others estimated aerobic fitness from field-based tests such as the Yo-Yo IR1 or Course Navette. The absence of significant effects in the Yo-Yo test should also be interpreted in light of the methodological heterogeneity of the included studies. The interventions differed substantially in terms of HIIT format, training volume, intensity prescription, intervention duration, and comparator conditions. Such variability may have influenced the magnitude of training adaptations and contributed to the inconsistency of findings across studies. Furthermore, several interventions were relatively short in duration, and some studies included highly trained or elite youth players, potentially limiting the scope for further improvement in cardiorespiratory outcomes (Helgerud et al., 2001). Finally, the relatively small number of available studies and participants, together with the high risk of bias identified in several trials, reduces the certainty of the current evidence regarding this outcome.

An additional consideration when interpreting these findings is the nature of the comparator interventions. Several studies did not compare HIIT against passive controls, but rather against other active training approaches, including small-sided games, transition small-sided games, continuous endurance training, and high-volume training protocols. This distinction is important because many of these training methods are themselves capable of improving cardiorespiratory fitness through repeated exposure to moderate-to-high physiological demands (Buchheit and Laursen, 2013a). For example, small-sided games have been consistently associated with improvements in aerobic fitness in soccer players due to their intermittent high-intensity nature and sport-specific movement demands (Los Arcos et al., 2015). Consequently, the absence of significant between-group differences in some individual studies may not necessarily indicate a lack of effectiveness of HIIT, but rather that both interventions were capable of eliciting favorable adaptations (Los Arcos et al., 2015). This interpretation is partially supported by the present meta-analysis, where HIIT demonstrated a significant advantage for VO_2_max compared with active comparator/control conditions, but only a small effect when compared specifically with small-sided games. Therefore, the effectiveness of HIIT should be interpreted relative to the comparator employed, as different training approaches may produce comparable improvements in certain cardiorespiratory outcomes among youth soccer players.

The developmental characteristics of youth players should also be considered when interpreting these findings. During adolescence and the transition to early adulthood, physiological adaptations are influenced not only by training but also by biological maturation, growth-related changes in body composition, and neuromuscular development (Armstrong and Barker, 2011; Armstrong and Welsman, 2000; Armstrong et al., 1991). As a result, the interpretation of training-related adaptations in youth populations may be more complex than in adult athletes (Buchheit and Laursen, 2013a). This consideration may be particularly relevant when comparing the magnitude and consistency of findings across studies involving participants at different stages of maturation. Overall, the present findings support the use of HIIT as a strategy associated with improvements in VO_2_max in male under-20 soccer players. However, similar effects were not observed for Yo-Yo test performance, suggesting that the effects of HIIT may differ according to the cardiorespiratory outcome selected. Consequently, practitioners and researchers should carefully consider the specific outcome measures used when evaluating the effectiveness of HIIT interventions in youth soccer players.

### 4.2 Jump performance

This systematic review found that the evidence available does not show that vertical jump performance consistently improves after HIIT compared to control conditions in adolescent male soccer players. Throughout the studies included changes in the height of the jump were usually minimal and inconsistent, and there was no evident suggestion that HIIT offers more advantages than conventional or active training methods. These findings are consistent with previous reviews in soccer populations reporting trivial-to-small effects of HIIT on vertical jump performance compared with both passive and active control conditions (Clemente et al., 2021a; Kunz et al., 2019). From a physiological perspective, this lack of clear improvement is not unexpected. The development of explosive vertical power requires high mechanical loading, stretch–shortening cycle (SSC) specificity, and maximal concentric intent, all of which are central to neuromuscular power adaptations (Cormie et al., 2011; Komi, 2000; Markovic and Mikulic, 2010). In contrast, most HIIT protocols primarily emphasize running-based activities and metabolic stress, leading predominantly to cardiovascular and glycolytic adaptations rather than the neuromechanical stimuli necessary to enhance maximal power output (Buchheit and Laursen, 2013a). Repeating high-intensity efforts may activate type II fibers and enhance fatigue resistance; however, the lack of targeted mechanical overload most probably prevents a significant result in vertical jump performance, an idea that is in agreement with the training specificity principle (Behm and Sale, 1993). Some studies included changes of direction and deceleration that might have indirectly stimulated the SSC function; nevertheless, these components would have had little overload or movement specificity to bring about large gains in CMJ, SJ, and DJ height. In addition, the differences in the design of the protocols, duration of the interventions, weekly training load, and the outcome measures used between studies make it difficult to understand the results. This is also true for the limited number of studies that assess certain jump variables. Taking into account the small sample sizes and methodological inconsistency of the studies, the current literature is not in favor of HIIT as a single method for enhancing vertical jump in adolescent soccer players, and power-oriented training approaches that are more focused should be considered for explosive performance as the main goal.

It is noteworthy that some studies reported modest improvements in CMJ performance following HIIT, suggesting that certain protocol characteristics may hold practical relevance for adolescent soccer players. This tendency could be associated with the inclusion of high-demand eccentric actions such as decelerations, braking phases, and changes of direction within several HIIT formats, which may stimulate type II fiber recruitment and increase musculotendinous stiffness in the lower limbs, indirectly supporting stretch–shortening cycle efficiency (Buchheit and Laursen, 2013a; Duchateau and Amiridis, 2023; Hung et al., 2025; Suchomel et al., 2019). Nevertheless, the absence of consistent improvements across studies likely reflects the limited mechanical specificity of the applied HIIT protocols. The development of explosive power requires targeted mechanical overload, high movement velocity with maximal intent, and specific stretch–shortening cycle stimulation, all of which are central to vertical jump enhancement (Brearley and Bishop, 2019; Buchheit and Laursen, 2013a; Duchateau and Amiridis, 2023; Hung et al., 2025; Suchomel et al., 2019). Although HIIT can induce favorable neuromuscular and metabolic adaptations, its primary emphasis on intermittent running and metabolic stress does not fully reproduce the biomechanical and neuromuscular demands of vertical jumping, thereby restricting its capacity to produce clear and transferable improvements in jump height (Brearley and Bishop, 2019). In line with the principle of training specificity, several authors have highlighted that when the objective is to improve jump performance, training programs should incorporate plyometric or jump-based exercises that directly target rapid force production and stretch–shortening cycle mechanics, which may also enhance fatigue resistance during repeated explosive efforts and facilitate a more direct mechanical transfer to performance (Brearley and Bishop, 2019; Buchheit and Laursen, 2013a; Duchateau and Amiridis, 2023; Hung et al., 2025; Suchomel et al., 2019). From a methodological standpoint, variability in intervention design, session structure, and training load across the included studies further complicates interpretation, and the limited number of trials assessing certain jump variables, together with modest sample sizes and risk of bias, reduces the certainty of the current evidence.

### 4.3 Sprint performance

The two sets of HIIT interventions did not result in a statistically significant difference in the 10-m sprint performance, but there was a significant improvement in the 30-m sprint performance. One possible reason for the lack of effects in the 10-m sprint is the specific mechanical factors that determine the athletes’ early acceleration. The initial acceleration phase (0–10 m) is mainly dependent on the athlete’s capability to produce a high level of horizontal force relative to the body mass and to apply this force efficiently within very short ground contact times, which is explained in the force-velocity mechanical framework of sprinting (Morin et al., 2012). Hence, this phase’s improvement is most likely linked to neural adaptations, such as increased motor unit recruitment, firing frequency, and intermuscular coordination, which are typically achieved by heavy resistance training and plyometric interventions rather than by metabolic-oriented conditioning programs (Behm and Sale, 1993; Brearley and Bishop, 2019; Buchheit and Laursen, 2013a; Cormie et al., 2011; Duchateau and Amiridis, 2023; Hung et al., 2025; Morin et al., 2012; Suchomel et al., 2019). HIIT can include bouts of high-speed running, but it does not regularly aim at maximal horizontal force production or rate of force development needed for explosive acceleration, which is one of the reasons why there were no significant changes over very short sprint distances.

The notable gains over 30 m indirectly imply that HIIT has the potential to improve physiological characteristics related to the maximal velocity and speed maintenance phases. High-intensity interval training is known to create remarkable changes in glycolytic enzyme activity, phosphocreatine resynthesis capacity, buffering capacity, and mitochondrial density, which translate into better energy turnover during repeated high-intensity efforts (Buchheit and Laursen, 2013a). Such changes may help the athlete keep higher running speeds even after the initial acceleration. Moreover, sprint interval training (SIT) has been linked to enhanced running performance as well as increased ability to resist fatigue in trained athletes (Koral et al., 2018). Thus, the idea that repeated high-intensity efforts (RHE) can improve the capacity to maintain velocity finds support in the abovementioned studies. To sum up, the research indicates that HIIT may not provide a high enough level of neuromuscular stimulation to be able to improve early acceleration; however, it seems to be quite good at elevating the metabolic and fatigue resistance factors that underlie performance in longer sprint distances.

### 4.4 Limitations and strengths

The findings of this systematic review should be interpreted considering several limitations. Firstly, the total evidence base that could be obtained was quite limited (n=8 studies) and there were notable differences between the studies in terms of study design, HIIT protocols, outcome measures, as well as participant characteristics. Such methodological differences make it very hard to compare the results of the studies directly and lower the capacity of the review to provide definite evidence about the efficacy of HIIT in this population. Furthermore, a number of studies were considered to carry at least a moderate level of risk of bias and had a very small number of participants. These factors may have resulted in the reported outcomes falling short of the expected internal validity. All the interventions in the studies that were included here were fairly short in duration (≤12 weeks). This makes it impossible to gain an understanding of the lasting effects of HIIT in adolescence from the studies. Lastly, the fact that there was incomplete reporting of training intensity in some studies makes it difficult to figure out the relationships between the potential dose and the response. Another significant caveat that must be noted is that the selection only of male subjects is a major limitation, as the results of this review cannot speak for female athletes. Furthermore, since in most studies there was no control for biological maturation, growth-related variability during this developmental stage could be a source of confounding.

The review is, however, fortified by a number of strength: (i) it rigorously adhered to a well-defined, transparent, and auditable process demonstrated by compliance with the PRISMA declaration, successful registration ahead of time, comprehensive assessment of methodological quality, and the risk of bias being carried out with validated tools; (ii) conducted an extensive search that covered six databases representing different disciplines which overall contributes greatly to the literature being exhaustively identified; (iii) limited itself to the study population of male adolescent soccer players thereby filling a gap in the literature of HIIT, where the population of male adolescent soccer players is not very well represented and (iv) looked at the effects of HIIT from four different perspectives cardiorespiratory fitness, sprint performance and jump performance, thus yielding a multifactorial account of how HIIT might affect the integrated physical fitness profile in adolescence.

### 4.5 Practical applications

The results of this systematic review make subtle practical implications to the coaches and practitioners working with male adolescent soccer players. According to the available evidence, sprint performance within 30 meters could be improved through HIIT interventions; however, no consistent benefits were noted in cardiorespiratory fitness, especially in VO_2_max and Yo-Yo Intermittent Recovery Test performance. Although this may seem contradicting considering the cardiovascular nature of HIIT, these results should be cautiously interpreted, and a more targeted training approach should be employed for this age group.

One of the factors that may explain the no or minimal changes in VO_2_max is the inconsistencies in the methodology and context in which the studies were conducted. The majority of the papers we analyzed were of moderate to high risk of bias, had small samples, and different HIIT protocols (e.g., short-interval, long-interval, small-sided games). This kind of methodological heterogeneity could be the reason for the conflicting findings and might have hidden the possible physiological adaptations. Besides, the use of different methods and procedures for intensity monitoring and reporting makes it difficult to confirm if athletes were able to consistently reach the intensity levels needed to cause central cardiovascular adaptations.

In terms of physiology, substantial gains in VO_2_max depend largely on central adaptations, such as an increase in stroke volume, maximal cardiac output, and plasma volume expansion. These changes typically occur when there is enough accumulated time spent at high intensities, which is usually at or above 90% of VO_2_max or HRmax, along with a proper total cardiovascular load. Nevertheless, a number of the studies included in this review were quite brief (commonly 5, 8 weeks), and it cannot be guaranteed that there was enough exposure to the above-mentioned high-intensity levels to produce the desired effect. This could partly account for the lack of regular improvements, especially in young players who reportedly have average fitness levels and therefore to get further central adaptations, a more specific or prolonged stimulus might be necessary. In addition, having an active control group that was also given structured training of similar intensity may have left less room for differences between training methods to be detected.

Hence, those professionals who want to enhance the cardiorespiratory fitness (VO_2_max) of adolescent male soccer players through practice only, may find that just implementing a general HIIT protocol is not enough. It is very important that carefully planned HIIT sessions:

- Focus on a total time of at least 90% of VO_2_max or HRmax (Buchheit and Laursen, 2013a) during which the training intensity is very high and maintained (> 90% VO2max or HRmax) to cause central adaptations. This may be done by longer work intervals, shorter recovery periods, or more sessions per week depending on the athlete’s adaptability.
- Take into account prolonged intervention periods of more than the usual 8–12 weeks to permit the physiological changes adequate time to get established.
- Carefully keep track of and report training intensity and volume to ensure that the “dose” is accurately targeted at cardiorespiratory adaptations.
- Take into consideration the athlete’s biological maturation and training status. During adolescence, players experience rapid growth and development and their adaptation abilities differ widely. A stimulus which may be enough for an untrained person might be insufficient for a trained adolescent athlete.

Besides cardiorespiratory fitness, the use of HIIT formats that accurately simulate the specific sport-movement demands; e.g., change-of-direction drills, SSGs, or plyometric-based HIIT protocols is also suggested to practitioners by this paper. Even though these approaches may mainly work on jump performance and explosive actions, at the same time, they can be used to maintain or even increase the cardiac stimulus. Furthermore, the combination of HIIT with other training modes such as strength and plyometrics is highly recommended to increase players’ neuromuscular and metabolic performance components comprehensively so that they are well-prepared for the various physical demands of soccer.

## 5 Conclusion

This systematic review gathered and analyzed studies that delved into how HIIT impacts the physical performance of adolescent soccer players. When taken together, the studies presented in this review generally indicate that HIIT programs can help in enhancing various aspects of physical fitness such as aerobic endurance, speed and vertical jump among others. These findings emphasize the potential benefit of adding HIIT to the regular training schedule of youth soccer players not only for improving performance-related outcomes but also as a very efficient way of using the training time. Nevertheless, one should be careful when making generalizations from these results as there have been significant differences in terms of the training schemes, length of the interventions, and ways of measuring the outcomes in the various studies that were included in this review. Going forward, it would be ideal for researchers to use more uniform HIIT protocols and recruit bigger groups of participants so as to produce more reliable findings and also gain insights into the effects of HIIT in youth soccer players over a longer period of time.

## Data Availability

The original contributions presented in the study are included in the article/supplementary material. Further inquiries can be directed to the corresponding author.
