# Supplementary material for: Effects of high-intensity interval training on cardiorespiratory fitness, jump, and sprint performance in male under-20 soccer players: a systematic review
Source: Front Physiol. 2026 Jul 30;17:1828517. doi: 10.3389/fphys.2026.1828517 (PMC13467764; doi:10.3389/fphys.2026.1828517)
Supplement: Supplementary file 1 [file DataSheet1.pdf]

# Additional file. Search strategy for each database

**Review title:** Effects of high-intensity interval training on cardiorespiratory fitness, jump, and sprint performance in male under-20 soccer players: A systematic review

## PICOS framework

| PICOS element | Concept | Search application |
|---|---|---|
| P, Population | Male under-20 soccer players | soccer, football, adolescent, youth, junior, academy, U20-U15, under 20-under 15 |
| I, Intervention | High-intensity interval training and related soccer-specific interval formats | high-intensity interval training, HIIT, high-intensity intermittent training, interval training, small-sided games, sprint interval training, repeated sprint training, speed endurance training |
| C, Comparator | Active control or alternative training condition | control group, active control, training comparison (used mainly during screening, not necessarily as a mandatory search block) |
| O, Outcomes | Cardiorespiratory fitness, sprint, jump, agility, and related physical fitness outcomes | VO2max, VO2 max, VO2peak, Yo-Yo, Yo-Yo IR1, sprint, speed, vertical jump, countermovement jump, squat jump, drop jump, agility |
| S, Study design | Randomized controlled trials and intervention studies | randomized, randomised, trial, intervention, control group, controlled trial (optional narrowing block if needed) |

## PUBMED

| Step | Search string |
|---|---|
| **S1** | "soccer"[Title/Abstract] OR "football"[Title/Abstract] |
| **S2** | "high-intensity interval training"[Title/Abstract] OR "HIIT"[Title/Abstract] OR "high-intensity intermittent training"[Title/Abstract] OR "interval training"[Title/Abstract] OR "small-sided games"[Title/Abstract] OR "small-sided game"[Title/Abstract] OR "sprint interval training"[Title/Abstract] OR "repeated sprint training"[Title/Abstract] OR "speed endurance training"[Title/Abstract] |
| **S3** | "physical fitness"[Title/Abstract] OR "physical performance"[Title/Abstract] OR "cardiorespiratory fitness"[Title/Abstract] OR "aerobic fitness"[Title/Abstract] OR "VO2max"[Title/Abstract] OR "VO2 max"[Title/Abstract] OR "VO2peak"[Title/Abstract] OR "Yo-Yo"[Title/Abstract] OR "Yo-Yo IR1"[Title/Abstract] OR "agility"[Title/Abstract] OR "sprint"[Title/Abstract] OR "sprints"[Title/Abstract] OR "sprint performance"[Title/Abstract] OR "speed"[Title/Abstract] OR "vertical jump"[Title/Abstract] OR "countermovement jump"[Title/Abstract] OR "squat jump"[Title/Abstract] OR "drop jump"[Title/Abstract] OR "reactive strength"[Title/Abstract] |
| **S4** | "adolescent"[Title/Abstract] OR "adolescents"[Title/Abstract] OR "adolescence"[Title/Abstract] OR "youth"[Title/Abstract] OR "teen"[Title/Abstract] OR "teens"[Title/Abstract] OR "teenager"[Title/Abstract] OR "teenagers"[Title/Abstract] OR "junior"[Title/Abstract] OR "academy"[Title/Abstract] OR "U20"[Title/Abstract] OR "U19"[Title/Abstract] OR "U18"[Title/Abstract] OR "U17"[Title/Abstract] OR "U16"[Title/Abstract] OR "U15"[Title/Abstract] OR "under 20"[Title/Abstract] OR "under 19"[Title/Abstract] OR "under 18"[Title/Abstract] OR "under 17"[Title/Abstract] OR "under 16"[Title/Abstract] OR "under 15"[Title/Abstract] |
| **S5** | S1 AND S2 AND S3 AND S4 |

## MEDLINE (EBSCOhost)

| Step | Search string |
|---|---|

| | |
|---|---|
| **S1** | TI (soccer OR football) OR AB (soccer OR football) |
| **S2** | TI ("high-intensity interval training" OR HIIT OR "high-intensity intermittent training" OR "interval training" OR "small-sided games" OR "small-sided game" OR "sprint interval training" OR "repeated sprint training" OR "speed endurance training") OR AB ("high-intensity interval training" OR HIIT OR "high-intensity intermittent training" OR "interval training" OR "small-sided games" OR "small-sided game" OR "sprint interval training" OR "repeated sprint training" OR "speed endurance training") |
| **S3** | TI ("physical fitness" OR "physical performance" OR "cardiorespiratory fitness" OR "aerobic fitness" OR VO2max OR "VO2 max" OR VO2peak OR "Yo-Yo" OR "Yo-Yo IR1" OR agility OR sprint OR sprints OR "sprint performance" OR speed OR "vertical jump" OR "countermovement jump" OR "squat jump" OR "drop jump" OR "reactive strength") OR AB ("physical fitness" OR "physical performance" OR "cardiorespiratory fitness" OR "aerobic fitness" OR VO2max OR "VO2 max" OR VO2peak OR "Yo-Yo" OR "Yo-Yo IR1" OR agility OR sprint OR sprints OR "sprint performance" OR speed OR "vertical jump" OR "countermovement jump" OR "squat jump" OR "drop jump" OR "reactive strength") |
| **S4** | TI (adolescent OR adolescents OR adolescence OR youth OR teen OR teens OR teenager OR teenagers OR junior OR academy OR U20 OR U19 OR U18 OR U17 OR U16 OR U15 OR "under 20" OR "under 19" OR "under 18" OR "under 17" OR "under 16" OR "under 15") OR AB (adolescent OR adolescents OR adolescence OR youth OR teen OR teens OR teenager OR teenagers OR junior OR academy OR U20 OR U19 OR U18 OR U17 OR U16 OR U15 OR "under 20" OR "under 19" OR "under 18" OR "under 17" OR "under 16" OR "under 15") |
| **S5** | S1 AND S2 AND S3 AND S4 |

## CINAHL Complete (EBSCOhost)

| **Step** | **Search string** |
|---|---|
| **S1** | TI (soccer OR football) OR AB (soccer OR football) |
| **S2** | TI ("high-intensity interval training" OR HIIT OR "high-intensity intermittent training" OR "interval training" OR "small-sided games" OR "small-sided game" OR "sprint interval training" OR "repeated sprint training" OR "speed endurance training") OR AB ("high-intensity interval training" OR HIIT OR "high-intensity intermittent training" OR "interval training" OR "small-sided games" OR "small-sided game" OR "sprint interval training" OR "repeated sprint training" OR "speed endurance training") |
| **S3** | TI ("physical fitness" OR "physical performance" OR "cardiorespiratory fitness" OR "aerobic fitness" OR VO2max OR "VO2 max" OR VO2peak OR "Yo-Yo" OR "Yo-Yo IR1" OR agility OR sprint OR sprints OR "sprint performance" OR speed OR "vertical jump" OR "countermovement jump" OR "squat jump" OR "drop jump" OR "reactive strength") OR AB ("physical fitness" OR "physical performance" OR "cardiorespiratory fitness" OR "aerobic fitness" OR VO2max OR "VO2 max" OR VO2peak OR "Yo-Yo" OR "Yo-Yo IR1" OR agility OR sprint OR sprints OR "sprint performance" OR speed OR "vertical jump" OR "countermovement jump" OR "squat jump" OR "drop jump" OR "reactive strength") |
| **S4** | TI (adolescent OR adolescents OR adolescence OR youth OR teen OR teens OR teenager OR teenagers OR junior OR academy OR U20 OR U19 OR U18 OR U17 OR U16 OR U15 OR "under 20" OR "under 19" OR "under 18" OR "under 17" OR "under 16" OR "under 15") OR AB (adolescent OR adolescents OR adolescence OR youth OR teen OR teens OR teenager OR teenagers OR junior OR academy OR U20 OR U19 OR U18 OR U17 OR U16 OR U15 OR "under 20" OR "under 19" OR "under 18" OR "under 17" OR "under 16" OR "under 15") |
| **S5** | S1 AND S2 AND S3 AND S4 |

## SPORTDiscus (EBSCOhost)

| Step | Search string |
|---|---|
| **S1** | TI (soccer OR football) OR AB (soccer OR football) |
| **S2** | TI ("high-intensity interval training" OR HIIT OR "high-intensity intermittent training" OR "interval training" OR "small-sided games" OR "small-sided game" OR "sprint interval training" OR "repeated sprint training" OR "speed endurance training") OR AB ("high-intensity interval training" OR HIIT OR "high-intensity intermittent training" OR "interval training" OR "small-sided games" OR "small-sided game" OR "sprint interval training" OR "repeated sprint training" OR "speed endurance training") |
| **S3** | TI ("physical fitness" OR "physical performance" OR "cardiorespiratory fitness" OR "aerobic fitness" OR VO2max OR "VO2 max" OR VO2peak OR "Yo-Yo" OR "Yo-Yo IR1" OR agility OR sprint OR sprints OR "sprint performance" OR speed OR "vertical jump" OR "countermovement jump" OR "squat jump" OR "drop jump" OR "reactive strength") OR AB ("physical fitness" OR "physical performance" OR "cardiorespiratory fitness" OR "aerobic fitness" OR VO2max OR "VO2 max" OR VO2peak OR "Yo-Yo" OR "Yo-Yo IR1" OR agility OR sprint OR sprints OR "sprint performance" OR speed OR "vertical jump" OR "countermovement jump" OR "squat jump" OR "drop jump" OR "reactive strength") |
| **S4** | TI (adolescent OR adolescents OR adolescence OR youth OR teen OR teens OR teenager OR teenagers OR junior OR academy OR U20 OR U19 OR U18 OR U17 OR U16 OR U15 OR "under 20" OR "under 19" OR "under 18" OR "under 17" OR "under 16" OR "under 15") OR AB (adolescent OR adolescents OR adolescence OR youth OR teen OR teens OR teenager OR teenagers OR junior OR academy OR U20 OR U19 OR U18 OR U17 OR U16 OR U15 OR "under 20" OR "under 19" OR "under 18" OR "under 17" OR "under 16" OR "under 15") |
| **S5** | S1 AND S2 AND S3 AND S4 |

## SCOPUS

## Scopus Basic Search blocks

| Step | Search string |
|---|---|
| **S1** | soccer OR football |
| **S2** | "high-intensity interval training" OR HIIT OR "high-intensity intermittent training" OR "interval training" OR "small-sided games" OR "small-sided game" OR "sprint interval training" OR "repeated sprint training" OR "speed endurance training" |
| **S3** | "physical fitness" OR "physical performance" OR "cardiorespiratory fitness" OR "aerobic fitness" OR VO2max OR "VO2 max" OR VO2peak OR "Yo-Yo" OR "Yo-Yo IR1" OR agility OR sprint OR sprints OR "sprint performance" OR speed OR "vertical jump" OR "countermovement jump" OR "squat jump" OR "drop jump" OR "reactive strength" |
| **S4** | adolescent OR adolescents OR adolescence OR youth OR teen OR teens OR teenager OR teenagers OR junior OR academy OR U20 OR U19 OR U18 OR U17 OR U16 OR U15 OR "under 20" OR "under 19" OR "under 18" OR "under 17" OR "under 16" OR "under 15" |
| **S5** | S1 AND S2 AND S3 AND S4 |

## WEB OF SCIENCE CORE COLLECTION

## Web of Science Fielded Search / Query Builder blocks

| Step | Search string |
|---|---|
| **S1** | soccer OR football |

| | |
|---|---|
| **S2** | "high-intensity interval training" OR HIIT OR "high-intensity intermittent training" OR "interval training" OR "small-sided games" OR "small-sided game" OR "sprint interval training" OR "repeated sprint training" OR "speed endurance training" |
| **S3** | "physical fitness" OR "physical performance" OR "cardiorespiratory fitness" OR "aerobic fitness" OR VO2max OR "VO2 max" OR VO2peak OR "Yo-Yo" OR "Yo-Yo IR1" OR agility OR sprint OR sprints OR "sprint performance" OR speed OR "vertical jump" OR "countermovement jump" OR "squat jump" OR "drop jump" OR "reactive strength" |
| **S4** | adolescent OR adolescents OR adolescence OR youth OR teen OR teens OR teenager OR teenagers OR junior OR academy OR U20 OR U19 OR U18 OR U17 OR U16 OR U15 OR "under 20" OR "under 19" OR "under 18" OR "under 17" OR "under 16" OR "under 15" |
| **S5** | S1 AND S2 AND S3 AND S4 |
